# Bidirectional Communication Between the Innate and Adaptive Immune Systems

**DOI:** 10.1146/annurev-immunol-083122-040624

**Published:** 2025-04

**Authors:** Kathrynne A. Warrick, Charles N. Vallez, Hannah E. Meibers, Chandrashekhar Pasare

**Affiliations:** Division of Immunobiology and Center for Inflammation and Tolerance, Cincinnati Children’s Hospital Medical Center, Department of Pediatrics, College of Medicine, University of Cincinnati, Cincinnati, Ohio, USA

**Keywords:** T cell memory, TNF superfamily, sterile inflammation, autoimmunity, microbial defense, CAR-T cells, cytokine storms

## Abstract

Effective bidirectional communication between the innate and adaptive immune systems is crucial for tissue homeostasis and protective immunity against infections. The innate immune system is responsible for the early sensing of and initial response to threats, including microbial ligands, toxins, and tissue damage. Pathogen-related information, detected primarily by the innate immune system via dendritic cells, is relayed to adaptive immune cells, leading to the priming and differentiation of naive T cells into effector and memory lineages. Memory T cells that persist long after pathogen clearance are integral for durable protective immunity. In addition to rapidly responding to reinfections, memory T cells also directly instruct the interacting myeloid cells to induce innate inflammation, which resembles microbial inflammation. As such, memory T cells act as newly emerging activators of the innate immune system and function independently of direct microbial recognition. While T cell–mediated activation of the innate immune system likely evolved as a protective mechanism to combat reinfections by virulent pathogens, the detrimental outcomes of this mechanism manifest in the forms of autoimmunity and other T cell–driven pathologies. Here, we review the complexities and layers of regulation at the interface between the innate and adaptive immune systems to highlight the implications of adaptive instruction of innate immunity in health and disease.

## INTRODUCTION

**Figure 1:**
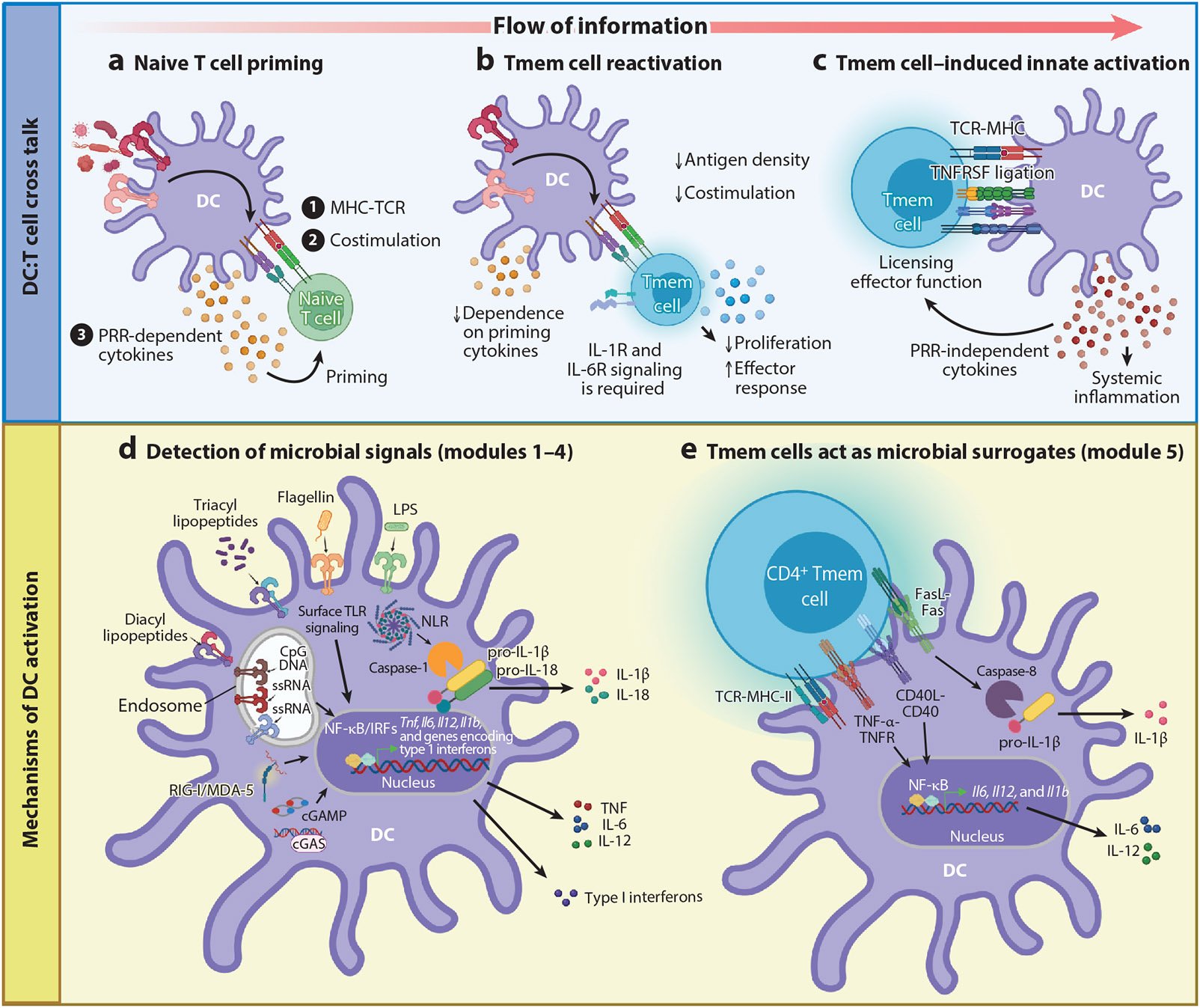
A comprehensive overview of the bidirectional communication between the innate and adaptive immune systems. (*a*) Naive T cell priming is a stringent process governed by DC activation, resulting from the detection of microbial signatures by PRRs. PRR signaling drives several DC cellular alterations, including ① increased antigen processing and the expression of MHC-peptide complexes on the cell surface, ② rapid upregulation of costimulatory molecules, and ③ production of cytokine cues that regulate T cell subset differentiation. (*b*) Tmem cell reactivation is a less rigid process that has a decreased requirement for both antigen density and costimulatory molecule engagement. Tmem reactivation is uncoupled from PRR signaling yet still depends on innate cytokines, such as IL-1 and IL-6, suggesting the involvement of alternative mechanisms of innate immune activation during cognate interactions between memory CD4^+^ T cells and DCs. (*c*) Tmem cells serve as microbial surrogates during cognate interactions with DCs. This interaction relies on TNFSF ligand expression by Tmem cells and results in the innate cytokine production necessary for the licensing of Tmem effector functions. (*d*) This overview shows modules 1–4 of DC microbial sensing mechanisms in various subcellular compartments that are strategically positioned to detect diverse pathogenic insults and initiate appropriate inflammatory responses. (*e*) In module 5 of innate activation, Tmem cells act as surrogate microbial signals through the illustrated molecular mechanisms. TNFSF ligand–receptor interactions induce NF-κB activation and caspase-8 cleavage in DCs to drive proinflammatory cytokine production (e.g., IL-6, IL-12, and IL-1β). Abbreviations: cGAMP, cyclic GMP-AMP; cGas, cyclic GMP-AMP synthase; DC, dendritic cell; IRF, interferon regulatory factor; LPS, lipopolysaccharide; MDA-5, melanoma differentiation–associated protein 5; NLR, NOD-like receptor; NOD, nucleotide-binding oligomerization domain–containing protein; PRR, pattern recognition receptor; RIG-I, retinoic acid–inducible gene I; TCR, T cell receptor; TLR, Toll-like receptor; Tmem, memory T; TNF, tumor necrosis factor; TNFR, TNF receptor; TNFRSF, TNFR superfamily; TNFSF, TNF superfamily. Panels *a* and *c* adapted from Reference ^15^ with permission from Elsevier. Panels *b*, *d*, and *e* adapted from images created with BioRender.com.

The innate and adaptive immune systems use distinct receptors and cells to protect the host from microbial invaders (1). Innate immune cells express germline-encoded receptors to detect conserved microbial structures and virulence factors (2). The recognition of microbial ligands by pattern recognition receptors (PRRs) culminates in the activation of cells and production of proinflammatory cytokines (1, 3). This response facilitates the elimination of the pathogen and subsequent activation of the adaptive immune system (4-6). The innate immune system is thus uniquely positioned to recognize, respond to, and eliminate microbial nonself. In contrast, somatically rearranged receptors on cells of the adaptive immune system lack predetermined specificity to the invading pathogen (1, 2). The innate immune system’s ability to distinguish self from microbial nonself generates specific cues that are then relayed to T cells and B cells (1, 6, 7). While many cell types express PRRs, the myeloid cells of the hematopoietic system, specifically macrophages and dendritic cells (DCs), play key roles in orchestrating innate and adaptive immune responses (7, 8). The activation of DCs by PRRs leads to the priming and differentiation of naive T cells via a tightly regulated process that requires three major signals emanating from DCs in the forms of MHC-peptide complexes, costimulatory molecules, and cytokines (9-11). Naive T cells that receive all three signals from pathogen-activated DCs undergo robust activation and clonal expansion to generate effector and memory T cells (6, 8). It has long been proposed that memory T cells need only MHC-peptide complex interaction for activation (12, 13). This less-stringent activation model is supported by evidence that memory T cell function is uncoupled from proximal activation of the innate immune system by microbial ligands (14-18). However, recent evidence suggests that CD4^+^ memory T cells still depend on cytokines and other cues produced by antigen-presenting cells (APCs) (19-22). The dependence of memory T cell function on innate cytokines but not on PRR signaling poses an interesting paradox: How are innate signals produced without microbial ligands? Several studies now propose that memory T cells act as microbial surrogates to drive innate immune activation and inflammation (23-28). While T cell–mediated activation of the innate immune system might have evolved as a protective mechanism to combat reinfections by virulent pathogens, negative outcomes, including autoimmunity, T cell–induced cytokine storms, and transplant rejection, arise. Here, we briefly review the classical flow of information from the innate immune system to the adaptive immune system. We also highlight the emerging mechanisms of innate immune activation by memory T cells that augment not only pathogen clearance but also inflammatory diseases (Figure 1).

## CLASSICAL TRANSFER OF INFORMATION: INNATE CONTROL OF ADAPTIVE IMMUNITY

### Antigen-Presenting Cells as Sensors of Tissue Disturbances

The innate immune system is composed of specialized cells that express unique receptors to recognize diverse microbial ligands. While their primary function is to mount a rapid inflammatory response that eliminates microbes, innate immune cells also encode and relay microbial information to educate the adaptive immune system. Briefly, APCs express germline-encoded receptors to detect conserved microbial structures and virulence factors. Since Charles Janeway Jr. (2) proposed the existence of PRRs for self-nonself discrimination, several families of PRRs, their ligands, and downstream signaling pathways have been elucidated (2, 29-33). Different PRRs convey specific information about the nature of the pathogen, leading to divergent responses optimally suited for each invading pathogen. These outcomes depend on multiple factors including the cell type, the subcellular location of PRRs, adaptor molecules to propagate signaling, ligand engagement timing and concentration, and the transcriptional and epigenetic state of the cells (34-36). PRR signaling underscores the defining feature of the innate immune system to recognize, respond to, and eliminate the microbial nonself while remaining largely inert to self-molecules. In addition to detecting microbial signals, APCs sense disturbances in cellular and tissue homeostasis (37-40). Chief among these perturbations are virulence factors, toxins, and products released following cellular damage and death. Other cues include metabolites of either host or microbial origin (41, 42). Thus, APCs constantly monitor their immediate environment to maintain homeostasis or to initiate inflammation upon sensing threats.

APC sensing can be classified into four core modules. The first module involves the classical recognition of conserved products of microbes, irrespective of their pathogenic capability. The primary sensing receptors in this module, which are predominately on hematopoietic innate cells, include the Toll-like receptor (TLR) family of receptors (33) and C-type lectin receptors, such as Dectin-1 and Dectin-2 (32). The second module is a cytosolic-sensing mechanism utilized by any cell targeted for microbial invasion and replication. Both hematopoietic and nonhematopoietic cells that are targets for viral and intracellular bacterial infections express cytosolic sensors NOD1 (nucleotide-binding oligomerization domain–containing protein 1) and NOD2 (43) and express nucleic acid–sensing receptors, such as RIG-I (retinoic acid–inducible gene I), MDA-5 (melanoma differentiation–associated protein 5), and cGAS (cyclic GMP-AMP synthase) (44). The diverse repertoire of receptor expression allows innate cells to mount an antimicrobial defense and to alert neighboring cells to infections. The classical PRR signaling described in the first two modules results in an inflammatory response defined by the secretion of innate cytokines, such as tumor necrosis factor (TNF), IL-6, and type I interferons that are targets of NF-κB, AP-1 (activator protein 1), and the interferon regulatory factor (IRF) family of transcription factors (45, 46). This response serves to contain and eliminate a variety of pathogens that have breached host defenses and invaded the tissues. A third module addresses the additional threat posed by pathogens expressing toxins or virulence factors. These pathogens require additional responses from the immune system, which is accomplished by the formation of a multiprotein signaling complex called the inflammasome (47-49). Inflammasome activation results in the cleavage and release of cytokines such as IL-1β and IL-18 and in pyroptotic cell death. For a more detailed overview of the various inflammasome complexes and their ligands, we refer our readers to other reviews (see 47 and 48). Lastly, the fourth module detects self-products that are released after cell damage or death. Several cytokines, such as IL-1α, IL-33, and TSLP (thymic stromal lymphopoietin), are made by epithelial cells and other stromal cells, and the former two are released following cell death (50, 51). These cytokines promote type I or type II inflammation, tissue repair processes, the elimination of pathogens, and the clearance of damaged cells (52-54) (Figure 1d).

### Dendritic Cells as a Bridge Between Innate and Adaptive Immunity

Microbial invasion and tissue disturbances are primarily sensed by tissue resident cells. Although PRR activation initiates essential inflammatory processes, these preliminary responses alone are often insufficient to fully combat infections. As such, the generation of pathogen-specific adaptive immunity represents a critical step in functional immunity. DCs are uniquely suited to bridge the innate and adaptive arms for several reasons. First, DCs are found within all tissues of the body and are highly phagocytic even in an immature state, allowing them to constantly sample their surroundings (55). Second, DCs express many PRRs to respond to diverse microbial cues and provide signals for T cell differentiation (6-8). Third, DCs can traffic to secondary lymphoid organs for engagement with naive T cells (56, 57). Finally, DCs avoid several forms of premature cell death (58-60), including the suppression of inflammasome machinery (61, 62), to ensure the transmission of information to the adaptive immune system (15, 61-64). Cumulatively, these characteristics underscore the pivotal role of DCs in linking innate and adaptive immunity.

The precise coordination of signals emanating from activated APCs occurs via a process defined as the three-signal paradigm. The three signals include MHC-peptide complexes, costimulatory molecules, and priming cytokines (9, 11). These signals are typically provided by DCs that have encountered pathogens, and thus are activated through PRRs (7, 8). This strict regulation of naive CD4^+^ T cell activation ensures that adaptive immune responses are mounted only against microbial-derived peptides, as opposed to self-antigens. In fact, the presentation of self-peptides, in the absence of additional signals, leads to T cell anergy (65). This regulated flow of information from the innate immune system to the adaptive immune system is thus integral to the generation of long-term protective immunity.

### Tailored Immunity and Cues Beyond the Three-Signal Paradigm

Multiple lineages of DCs have been characterized since their initial discovery by Ralph Steinman and Zanvil Cohn (66, 67). DCs are classified as conventional or classical DCs (cDC1 and cDC2), plasmacytoid DCs, monocyte-derived DCs, or Langerhans cells. DC subsets, differing in anatomical location and function, drive context-dependent differentiation of naive CD4^+^ T cells. For example, T helper 1 (Th1) and CD8^+^ T cells, both critical mediators of intracellular pathogens and tumor surveillance, are generally induced by cDC1s (68). Notably, Th1 polarization can be partially explained by higher IL-12 production in cDC1s compared with cDC2s (69, 70). On the other hand, cDC2s primarily drive CD4^+^ T cell priming (including Th2, Th17, and T follicular helper cell differentiation). Conditional disruption of cDC2s impairs the differentiation of these subsets across multiple disease settings (71-73). For a more comprehensive review of DCs and their ability to drive differential adaptive responses, we refer our readers to other reviews (see 68 and 74).

Despite these established roles of DC subsets, the traditional three-signal paradigm provides a limited explanation of how DCs sculpt an effective T cell response to specific pathogens, given that many PRRs induce broadly similar outcomes (35). One distinct possibility is that innate immune cells integrate additional information. Pathogen-specific and virulence factor–dependent changes to cellular homeostasis could also be incorporated with direct microbial cues. Additional mechanisms of homeostatic sensing, such as ER stress, nutrient availability, and cell-intrinsic metabolic alterations, are all likely to dictate the outcome of adaptive immune responses, including the elusive mechanisms driving Th2 and Th17 responses (75-77). The uncovering of the exact cues, outside of canonical sensing, represents an exciting area for future research.

## REVERSE FLOW OF INFORMATION: ADAPTIVE INSTRUCTION OF INNATE IMMUNITY

Immunological memory is a cardinal feature of the adaptive immune system. Following the elimination of the primary insult, memory T cells can be maintained for decades and are poised to respond rapidly to a secondary exposure. In contrast to the stringent rules for naive T cell priming, the reactivation of memory T cells is more permissive (78). For example, memory T cells proliferate at lower antigen concentrations and exhibit more rapid production of effector cytokines (e.g., IFN-γ, IL-4, and IL-5) (12, 78, 79). Beyond antigen density, memory T cells have unique T cell receptor (TCR) signaling downstream of TCR-MHC engagement. For example, TCR stimulation induces distinct phosphorylation of ERK (extracellular signal-regulated kinase) and p38 in naive versus antigen-experienced T cells (80). Specifically, the activation of naive cells favors the ERK pathway, which results in diminished calcium elevation. These findings suggest that biases in mitogen-activated protein kinase phosphorylation contribute to differences in naive and antigen-experienced T cell responses downstream of TCR ligation (80). Furthermore, the rapid induction of NF-κB transcriptional activity and increased engagement of NF-κB on the IFN-γ promoter were observed in memory T cells when compared with naive T cells following TCR stimulation (81). Thus, enhanced NF-κB activation and promoter engagement explains the rapid kinetics of TCR-mediated signaling during memory T cell reactivation.

While memory CD4^+^ T cells have less stringent requirements for reactivation, recent studies have also revealed that the optimal function of memory T cells, especially in vivo, still depends on innate cytokines and costimulatory molecules (12, 19, 21, 22, 82) (Figure 1b). It is both perplexing and intriguing that this dependence is uncoupled from proximal PRR sensing by APCs (14-17); this uncoupling suggests that there are alternative mechanisms of innate immune cell activation. During the reactivation of memory T cells, the immunological synapse allows for a complex bidirectional flow of information in the form of both soluble and membrane-bound molecules. While the molecular interactions underlying the innate to adaptive transfer of information have been well-characterized, mechanisms by which adaptive cells influence innate activation, especially in a cognate fashion, have received less attention. In this section, we review the signals emanating from memory T cells that influence innate immune function.

### Canonical Functions of Memory CD4^+^ T Cells

CD4^+^ T cells, together with CD8^+^ T cells, represent the majority of T lymphocytes. Unlike the cytotoxic activity characteristic of CD8^+^ T cells, the primary function of CD4^+^ T cells is to orchestrate the activity of other immune cells, as well as to exert some direct effector functions. These cells compose diverse subsets defined by distinct cytokine profiles, which are crucial for sustaining effective and regulated immune responses to pathogens. Importantly, CD4^+^ T cells modulate immune responses through both soluble factors and membrane-bound interactions with target cells. Here, we highlight the well-characterized roles of CD4^+^ T cells, including their contributions to B cell activation and the licensing of DCs to achieve optimal CD8^+^ T cell responses.

#### Soluble mediators.

The primary function of antigen-experienced CD4^+^ T cells, including effector and memory subsets, is to release soluble mediators that in turn mobilize and enhance functions of the innate immune system (52, 83, 84). Th1 cells combat intracellular pathogens through the secretion of the lineage-defining cytokine IFN-γ, as well as through IL-2 and TNF. Of these cytokines, IFN-γ is a potent activator of macrophages that drives polarization into a proinflammatory type 1 (M1) macrophage (85). IFN-γ also enhances macrophage responsiveness to stimuli, including TLR ligands and TNF, and IFN-γ stimulates antigen presentation by upregulating MHC molecules to further promote T cell activation (86).

The canonical Th2 cytokines including IL-4, IL-5, and IL-13 regulate tissue repair, protect from parasitic infections, and contribute to allergic diseases (87). IL-13 acts on the epithelium to induce mucin production for allergen elimination but also is known, along with IL-4, to skew the macrophage phenotype toward the alternatively activated (M2) phenotype (88). Th17 responses are characterized by neutrophil infiltration and have the potential for severe tissue destruction (89). The cardinal Th17 cytokines IL-17A and IL-17F induce the production of proinflammatory cytokines, granulopoiesis factors [granulocyte colony-stimulating factor (G-CSF) and stem cell factor], chemokines (CXCL1, CXCL2, and CXCL5), and matrix metalloproteinases by multiple cell types (90). The detailed characterization of each Th subset and the effects attributed to each cytokine profile are fully reviewed elsewhere (see 52). This brief overview provides examples of the classical understanding of how Th subsets direct the flow of information from the adaptive immune system to the innate immune system.

#### B cell help.

As outlined above, CD4^+^ T cells act as central anchors of functional immunity. However, the importance of memory CD4^+^ T cells in driving antimicrobial responses outside of cytokine production warrants attention. An important function of activated CD4^+^ T cells is to provide help to antigen-specific B cells. Primed effector and memory CD4^+^ T cells modulate B cell responses through cognate interactions as well as secreted mediators. It is widely accepted that CD40L on CD4^+^ T cells engages CD40 on antigen-experienced B cells to drive germinal center formation, immunoglobulin class switching, and affinity maturation (91, 92). Subsequently, the T cell–derived soluble mediators IFN-γ and IL-4 directly influence B cells by dictating class switching to IgG and IgE, respectively. While this function of CD4^+^ T cells has long been appreciated (93), below we focus on the more recent characterization of membrane-bound interactions between CD4^+^ T cells and cells of the innate immune system.

#### cDC1 licensing via cell-surface interactions.

Beyond the direct effects of cytokines, CD4^+^ T cells influence CD8^+^ T cells indirectly through DCs. In the absence of CD4^+^ T cells, DCs alone are generally insufficient to drive optimal CD8^+^ T cell responses, except in certain infections (94-97). Like B cell help, DC licensing depends on CD40L:CD40 engagement. Indeed, signaling through CD40, facilitated by CD40L expressed on CD4^+^ T cells, enables the cross-priming capacity of DCs to effectively stimulate CD8^+^ T cells (98, 99). This mechanism represents some of the first evidence that primed CD4^+^ T cells (effector and memory) essentially mimic microbial ligands, thereby allowing the conditioning of cDC1s to prime CD8^+^ T cells (100).

Similar interactions between primed CD4^+^ T cells and DCs are critical for effective antitumor immunity. Here, antigen-dependent interactions between CD4^+^ T cells and DCs lead to optimized antigen presentation, costimulatory molecule upregulation, and cytokine production by DCs; this cytokine production drives CD8^+^ T cell expansion and differentiation into effector and, subsequently, memory T cells (101). While the effects of CD40 signaling on DC antigen presentation and costimulatory molecule expression were known, direct stimulation of DCs with antibodies (in the absence of CD4^+^ T cells) clarified the role of DC-intrinsic CD40 signaling in supporting CD8^+^ T cell responses (100). Seminal work from the Murphy lab (102) demonstrated that CD4^+^ T cell–mediated licensing of cDC1s through CD40 was indispensable for the priming of antitumor CD8^+^ T cells. Notably, the requirements for CD4^+^ T cell activation to initiate this circuit remain unclear. In addition, Murphy and colleagues (103) identified a role for CD40L:CD40 in promoting cDC1 survival. The requirements of CD40 signaling for CD8^+^ T cell priming in both antimicrobial and tumor immunity share remarkable similarities. However, one important distinction is that microbes contain PAMPs (pathogen-associated molecular patterns) to elicit canonical innate signaling. In this context, the contribution of CD4^+^ T cell help may be redundant, amplifying, or complementary to signals received directly by DCs (101). Cancer cells, on the other hand, are not known to directly stimulate PRR signaling. Thus, we propose that in the absence of classical innate signals derived from microbial ligands, antitumor CD8^+^ T cell responses rely primarily on CD4^+^ T cell help. In essence, primed effector and memory CD4^+^ T cells act as microbial surrogates to activate cDC1s, which facilitate antitumor immunity. However, the fundamental question of how tumor-specific memory CD4^+^ T cells arise in the absence of necessary priming signals remains unresolved. One possibility is that nontraditional innate cues emanating from tumor cells activate DCs to induce the initial CD4^+^ priming, although this possibility remains an area of active investigation (104).

### Noncanonical Functions of CD4^+^ T Cells

The nonredundant role of CD4^+^ T cells in antimicrobial protection is well-established and is primarily attributed to their ability to produce cytokines and provide help to B cells. The extent of CD4^+^ T cell contribution to broader immunity through the involvement of additional arms of the immune system remains underexplored. Beyond their canonical functions, CD4^+^ T cells play a critical role in facilitating the activation of the innate immune system. In this section, we explore the contact-dependent mechanisms governing memory CD4^+^ T cell–driven innate inflammation.

#### Antiviral protection by memory CD4^+^ T cells.

Memory CD4^+^ T cells also promote immunity independently of effector cytokine production and canonical helper functions. Influenza infection studies conducted in healthy patients demonstrated that preexisting memory CD4^+^ T cells responded to influenza proteins of the challenge virus strain and correlated with decreased disease severity (105). More importantly, preexisting CD4^+^, but not CD8^+^, T cells responding to influenza internal proteins were associated with lower virus shedding and less severe illness (105). Critical work from Susan Swain and her group (27) using mouse models of influenza established a role for memory CD4^+^ T cells in protection against heterosubtypic strains of virus. Importantly, antigen-specific memory, but not naive, CD4^+^ T cells mediated early viral control (27). Since these initial observations, work from other groups has emphasized the importance of memory CD4^+^ T cell reactivation in protection from pathogens. Donna Farber’s group (106, 107) clarified the importance of tissue-resident memory (Trm) CD4^+^ T cells in eliciting similar protective responses. These results were consistent with reports by other researchers, who demonstrated that repeat immunizations to enhance Trm cell populations provide durable protection from mucosal infection even at suboptimal neutralizing antibody levels (108). Furthermore, CD4-mediated antiviral immunity can occur independently of both CD8^+^ T cell and B cell responses, effectively ruling out the canonical helper roles of CD4^+^ T cells (109).

#### Broad innate activation by memory CD4^+^ T cells.

While multiple mechanisms driving memory CD4^+^ T cell–mediated protection have been suggested, the ability of memory CD4^+^ T cells to induce broad innate immune activation is especially compelling (27). The pretreatment of mice with bacterial components to directly drive innate inflammation prior to viral infection resulted in improved survival, suggesting that early innate responses confer broad antimicrobial protection (110). In line with this finding, memory CD4^+^ T cells that were generated following a primary infection induced an acute increase in several key innate proinflammatory cytokines (e.g., IL-1β, IL-6, and IL-12) after influenza challenge (27, 111). Notably, the levels of these cytokines were much higher than those in naive mice that received the same challenge virus (27). The transfer of antigen-specific memory CD4^+^ T cells but not of naive CD4^+^ T cells before challenge led to high levels of inflammatory cytokines in the lungs of the recipient mice. More importantly, the depletion of CD4^+^ T cells significantly blunted the innate inflammatory response. This source of innate inflammation had significant impacts on viral control and was independent of classical Th1 cytokines (27), suggesting that there are alternative mechanisms of innate activation. Further, patients receiving vaccine boosters showed significantly elevated innate immune signatures after the second dose (112); this finding indicates that the formation of memory T cell responses may enhance innate immune responses upon subsequent antigen encounters.

#### Innate activation independent of pathogen sensing.

To delineate the effects of T cell–induced innate activation from classical virus-induced effects, Strutt et al. (27) transferred memory OT-II cells and infected hosts deficient in PAMP-sensing molecules (MyD88/TRIF signal adaptor proteins). Additionally, memory CD4^+^ T cells were reactivated via the administration of endotoxin-free ovalbumin protein following memory OT-II cell transfer. Innate cytokine production persisted in both settings, critically demonstrating independence from classical PRR signaling (27). This finding is especially provocative given the requirements for memory T cell reactivation. As alluded to in previous sections, direct microbial sensing by APCs is not an obligatory requirement for memory CD4^+^ T cell activation (17, 27). Despite the established lower stringency, memory CD4^+^ T cell reactivation still depends on innate cues. The most notable example of this is the involvement of innate cytokines during recall responses. The indispensable roles for innate cytokines during primary infection are well appreciated, but, more importantly, early IL-6 is paramount for the expansion of primed CD4^+^ T cells and enhances the production of IL-2, TNF, and IFN-γ during secondary infections (21).

To reconcile the apparent discrepancy between independence from PRR-mediated innate activation and persistent reliance on innate cues, we propose that the initial TCR engagement on memory T cells facilitates sufficient activation to engage DCs. Contact-mediated cognate interactions, demonstrated by the strict requirement of MHC-II expression on DCs, in turn activates DCs and increases their maturation status following memory T cell interaction (27). Our lab (28) also identified a requirement for TNF and FasL from memory T cells; TNF and FasL activate TNF receptor (TNFR) and Fas pathways in myeloid cells, leading to robust IL-1β synthesis and cleavage, respectively. On myeloid cells, signaling through TNFR and signaling through CD40 also synergize to induce IL-6 and IL-12 secretion (24). PRR-independent production of innate proinflammatory cytokines can then optimally drive the T cell response, leading to the amplification of inflammation. Taken together, these data suggest that memory CD4^+^ T cells effectively serve as microbial surrogates during anamnestic responses, facilitating rapid and broad innate activation that is vital for early protection from pathogens (Figure 1c,e).

### Role of the Tumor Necrosis Factor Superfamily in Innate-Adaptive Cross Talk

The TNF/TNFR superfamilies (TNFSF/TNFRSF) are composed of approximately 50 membrane and soluble proteins that contribute to numerous apoptotic, proliferative, and developmental processes (113, 114). Several receptors of the TNFSF are required for T cell activation. For a comprehensive review of costimulatory receptors, their ligands, and their roles in T cell activation, we refer readers to other reviews (see 113 and 115-118). Interestingly, there is significantly less focus on TNFRSF expression on myeloid cells and how the engagement of these receptors by ligands expressed on memory T cells could influence myeloid cell activation (119). DC subsets exhibit differential expression of CD40, TNFR, Fas, LTβR, HVEM, and RANK during maturation and in disease settings (120-122). Many of the TNFSF receptors expressed on DCs activate prosurvival signaling. Below, we highlight the roles of TNF and other TNFSF ligands expressed on T cell lineages and their contributions to innate inflammation through the engagement of cognate receptors on DCs.

#### T cell–derived tumor necrosis factor: a key trigger of innate inflammation.

Chief among the TNFSF members is the pioneer molecule TNF, which is common to multiple T cell subsets. While myeloid cell–derived TNF is an established product of innate immunity, the importance of T cell–derived TNF remains underappreciated (123-125). Following PRR activation, TNF is produced rapidly by several cells of the innate immune system, including macrophages and DCs (126). Acting in a pleiotropic manner, TNF propagates inflammation and drives additional processes such as apoptosis and necroptosis (123). Most of these functions are attributed to signaling through TNFR1, the primary receptor for soluble TNF, which is ubiquitously expressed by most cell types. T cells, including Th1, Th17, and CD8^+^ subsets, are also known to produce TNF. In fact, differentiated CD4^+^ T cells are specifically poised to make TNF by maintaining open chromatin conformation at the TNF transcription start site (127). This chromatin accessibility likely contributes to the significant impact of memory CD4^+^ T cell–derived TNF on protective responses. The same feature of memory CD4^+^ T cells also drives deleterious outcomes in the setting of autoimmunity. Differential sources of TNF have nonredundant roles in host immunity. Evidence for differential yet complementary roles was demonstrated in *Mycobacterium tuberculosis* infection, wherein early responses were mediated by TNF from myeloid cells while sustained protection was mediated by TNF from T cells (128). In line with this finding, mice deficient in T cell TNF had similar early responses to *Listeria monocytogenes* yet defective control at later time points (129). Interestingly, TNF can also be expressed by T cells in a transmembrane form that signals predominantly through TNFR2 (129). Soluble and membrane-bound TNF have distinct functions. For example, soluble TNF was critical for controlling primary infection with *Listeria*, while transmembrane TNF was sufficient to control secondary infection (130). Consistent with this finding, memory T cell transmembrane TNF facilitated the control of *Francisella tularensis* live vaccine strain (131). While therapeutic TNF inhibitors are commonly used in clinical practice, variable responses are likely due to the combined contributions of multiple cell sources as well as differential receptor signaling.

In the context of autoimmunity and alloimmunity, TNF produced by T cells correlated with adverse outcomes. In the experimental autoimmune encephalomyelitis model of multiple sclerosis, T cell–derived TNF mediated the infiltration of inflammatory myeloid cells into the central nervous system, resulting in worse autoimmune pathology (132). Similarly, in murine models of graft-versus-host disease driven by alloreactive donor T cells, recipients receiving grafts with TNF-deficient T cells had improved morbidity and mortality (133). Furthermore, the inhibition of TNF secretion by donor T cells significantly decreased graft-versus-host disease severity following allogeneic bone marrow transplantation (134). These studies highlight the importance of both membrane-bound and soluble TNF produced by memory T cells and demonstrate a critical role for this cytokine in T cell–mediated activation of the innate immune system for both protection and pathology.

#### Differential tumor necrosis factor superfamily ligand expression by T cell subsets.

**Figure 2:**
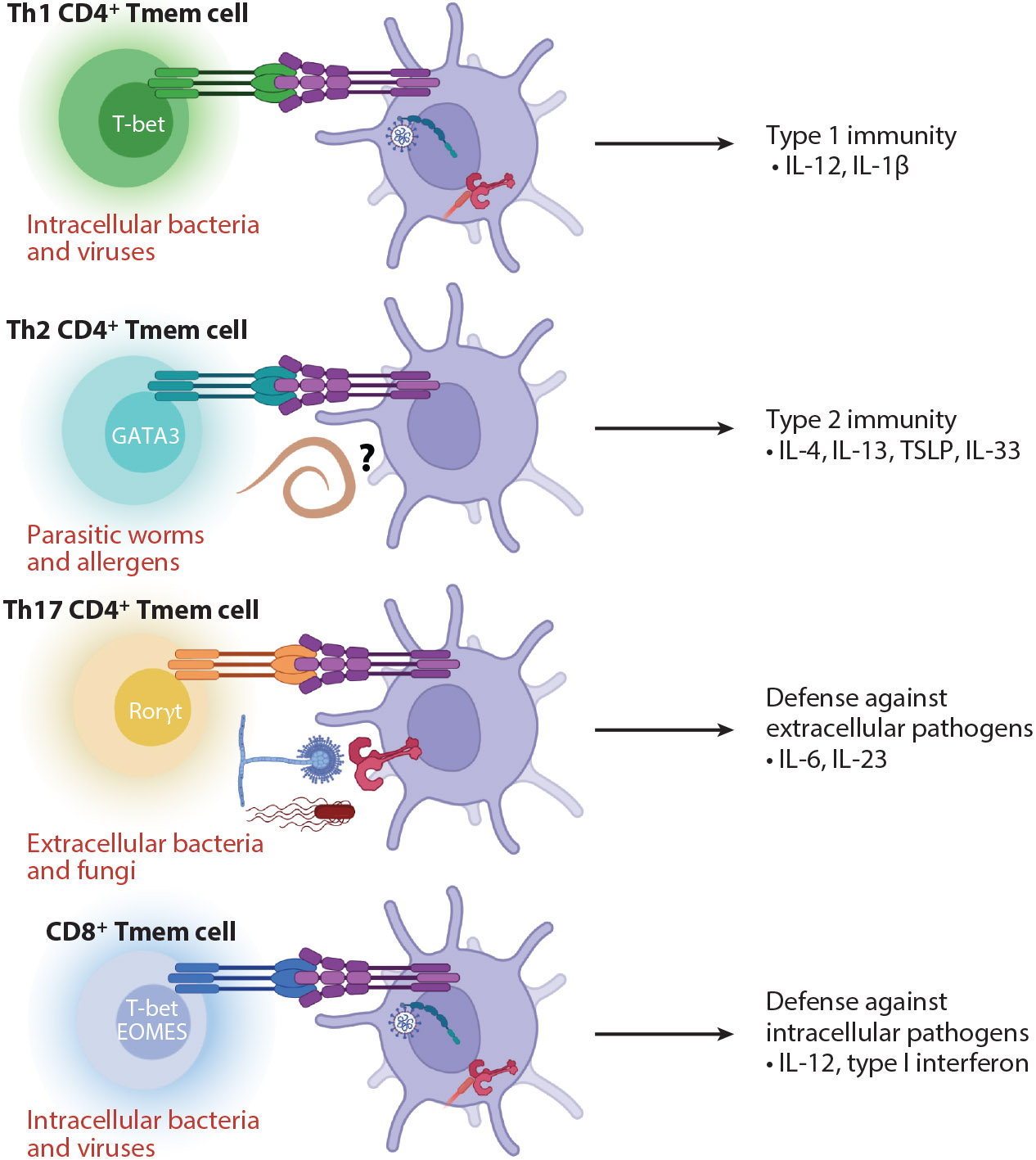
Memory CD4^+^ and CD8^+^ T cells act as microbial surrogates to promote highly specific inflammatory environments. The ability to induce differential innate inflammatory responses by different T cell lineages is dictated by the expression of specific TNFSF ligands by each subpopulation of primed T cells. Th1 CD4^+^ and CD8^+^ Tmem cells promote type 1 immunity that mirrors the innate sensing of intracellular pathogens. Th2 CD4^+^ Tmem cells promote type 2 immunity that parallels the innate sensing of allergens and helminth infections. Th17 CD4^+^ Tmem cells promote the production of cytokines necessary for defense against extracellular pathogens and for the maintenance of mucosal immunity. These highly specific inflammatory responses recapitulate those induced by the pathogens themselves to facilitate the tailored innate immune responses necessary for the rapid clearance of the pathogenic insult. Further, this system may serve as an evolutionary fail-safe against pathogens that are able to subvert PRR sensing. This mechanism ensures the production of critical innate inflammatory mediators, especially when previously primed Tmem cells or cross-reactive Tmem cells exist at the site of infection and in secondary lymphoid organs. Abbreviations: EOMES, eomesodermin; PRR, pattern recognition receptor; Th, T helper; Tmem, memory T; TNFSF, tumor necrosis factor superfamily; TSLP, thymic stromal lymphopoietin. Figure adapted from images created with BioRender.com.

Memory CD4^+^ subsets retain their originating effector cytokine profiles (79), but, more importantly, CD4^+^ Th subsets that are specific to the same antigen shape qualitatively different innate inflammatory responses (23, 135). Beyond differences in cytokines, there is sufficient experimental evidence to support the argument that differential expression of TNFSF molecules on T cell subsets drives TNFRSF signaling in myeloid cells to recapitulate features from the initial inflammatory setting upon a reencounter with the cognate antigen (Figure 2). This differential expression of TNFSF ligands, likely imprinted during the initial priming, manifests when memory T cells are reactivated to influence innate immune responses. Certain TNFSF ligands expressed on T cells, such as TNF and CD40L, are likely conserved across all Th populations, owing to their importance in several functions of helper T cells. We therefore posit that synergistic and combinatorial signaling of TNFRSF members on DCs shapes the innate immune response to provide optimal protection against the invading pathogen. In line with this proposed explanation, past work highlights the nuances in TNFSF ligand expression between Th populations. Th1 T cells are known to express RANKL (TRANCE) (136) and TRAIL, and the engagement of receptors for RANKL and TRAIL on DCs drove the production of IL-6, IL-12, and IL-15, cytokines associated with type 1 immunity (137). Further, T cell–derived RANKL signaling occurred in concert with TNF and CD40L, supporting the notion of cooperative signaling between different members of the TNFRSF (136). Th2 CD4^+^ T cells, in contrast, express higher levels of LIGHT. LIGHT signaling, through the receptor HVEM, has been shown to promote airway remodeling in ways resembling those of allergic asthma, including smooth muscle hyperplasia and pulmonary fibrosis (138). Patients with eosinophilic esophagitis, dominated by aberrant type 2 immunity, had a marked increase of LIGHT-expressing Th2 cells. Increased LIGHT expression correlated with the upregulation of IL-5 and IL-13 transcripts and mediated increased disease severity (139). TL1A (TNFSF15) has been shown to play a critical role in T cell costimulation and Th17 differentiation (140). Interestingly, T cells are also known to express TL1A, while the receptor, DR3, is expressed by many nonlymphocyte populations, including DCs and macrophages (141, 142). Polymorphisms in TL1A are associated with an enhanced macrophage proinflammatory cytokine signature in inflammatory bowel disease (IBD) (143). Additionally, the administration of TL1A neutralizing antibodies elicited endoscopic improvement in patients with moderate to severe ulcerative colitis (144). TL1A-deficient mice also exhibited decreased disease severity in collagen-induced arthritis without defects in Th17 differentiation or cytokine production (145). Thus, Th17-derived TL1A is a potential driver of autoimmunity and disease pathology. These studies support the notion that different T cell populations utilize disparate TNFSF ligand expression to influence innate immune responses. Given that myeloid populations express many TNFRSF members, the elucidation of how combinatorial ligand-receptor interactions facilitate innate immune responses may provide critical insights into the nuances of T cell–induced innate immune activation.

### Memory CD8^+^ T Cell Activation of the Innate Immune System

Canonically, CD8^+^ T cells combat intracellular pathogens and kill cancer cells by releasing cytotoxic granules. However, over the past 30 years, studies have also identified essential, noncytotoxic mechanisms through which CD8^+^ T cells contribute to protective immunity (146). Here, we focus on how these noncytotoxic mechanisms mobilize additional arms of the immune system and mediate protective responses through innate activation.

CD8^+^ T cells are known to secrete several soluble mediators that mobilize other cells of the immune system. The preeminent cytokine, IFN-γ, is rapidly secreted by memory CD8^+^ T cells upon a reencounter with antigen (147, 148). New lines of evidence suggest that IFN-γ secretion by CD8^+^ Trm cells also drives global antimicrobial responses in peripheral tissues through the induction of interferon-stimulated genes (149, 150). Further, these global responses appear sufficient to confer protection from antigenically distinct pathogens, suggesting that there is an evolutionary mechanism through which CD8^+^ Trm cells drive tissue-wide antimicrobial defenses upon reinfection (151). Despite the importance of IFN-γ, Jiang et al. (149) noted that several key antiviral genes upregulated upon memory CD8^+^ T cell reactivation were refractory to IFN-γ blockade, suggesting that additional signaling pathways contributed to this response.

One potential mediator of IFN-γ-independent function is TNF, which is secreted by both effector and memory CD8^+^ T cells. TNF has been implicated in the induction of type I interferon responses in an IRF1-dependent mechanism (152). In line with these findings, CD8^+^ T cells devoid of cytotoxic function were shown to elicit functional antilisterial immunity in an IFN-γ-independent but TNF-dependent mechanism (153). Another study has demonstrated that DC activation within the mucosal tissues depended on TNF production by CD8^+^ Trm cells (150). While TNF is a critical cytokine produced by CD8^+^ T cells, it is not the only known TNFSF ligand expressed by memory CD8^+^ T cells. Several other TNFSF ligands are now known to be expressed, including LTαβB, LIGHT, RANKL, TRAIL, and FasL. The stimulation of DCs by RANKL enhanced CD8^+^ T cell responses to tumor-associated antigens (154). Additional work implicated CD8^+^ T cell–derived RANKL as a driver of DC proinflammatory cytokine production (136). Another critical molecule in CD8^+^ T cell responses is 4–1BB (CD137). While the role of DC-derived 4-1BBL expression in CD8^+^ T cell activation has been well-established (155, 156), new data suggest that activated DCs also express 4-1BB and that signaling through this receptor elicited IL-6 and IL-12 production (157). These studies provide evidence that active and dynamic bidirectional communication between the innate and adaptive immune systems is a property shared by both CD4^+^ and CD8^+^ T cells.

## T CELL INSTRUCTION OF THE INNATE IMMUNE SYSTEM: IMPLICATIONS FOR HEALTH AND DISEASE

**Figure 3:**
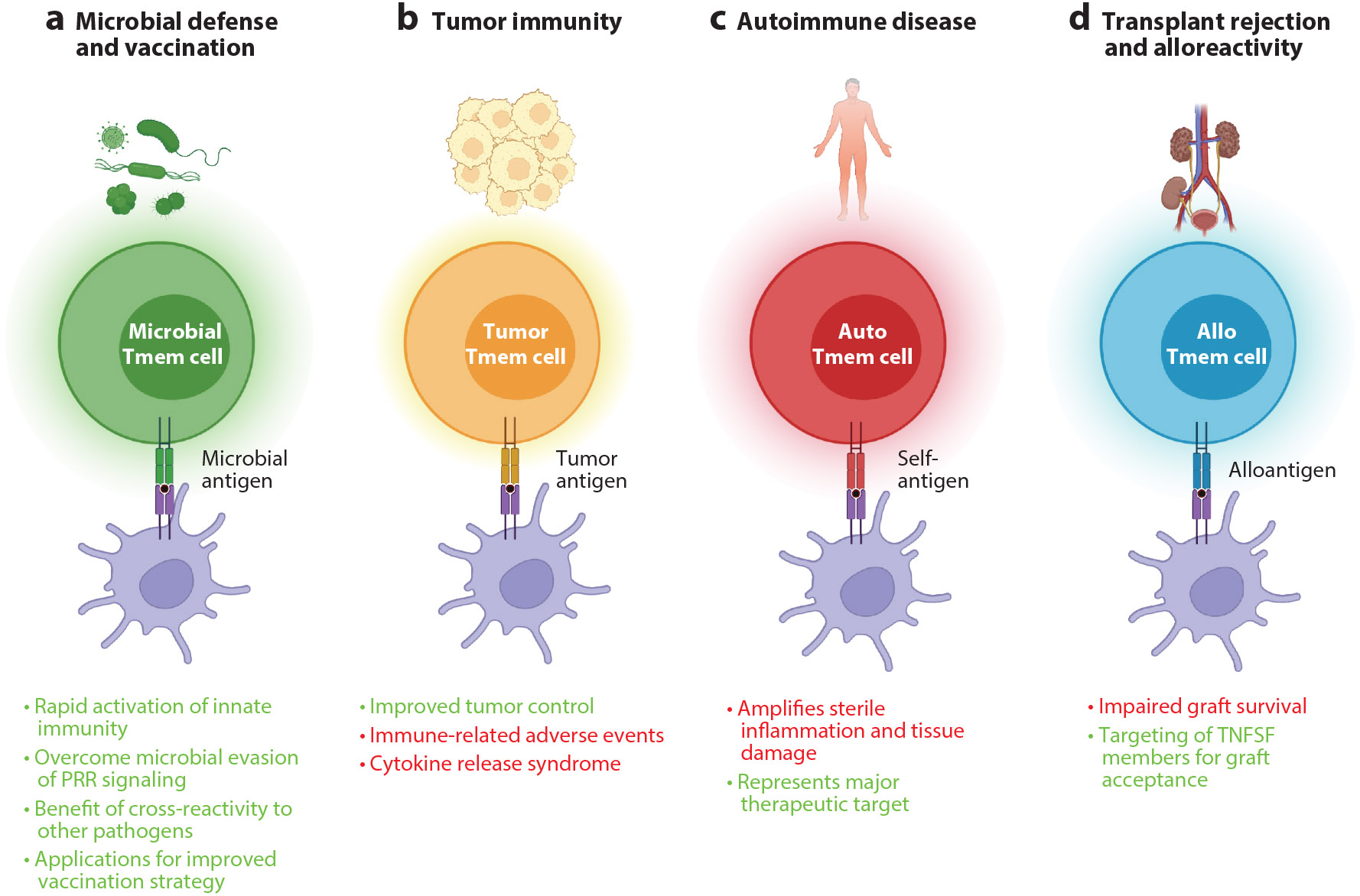
An overview of the beneficial (*green text*) and deleterious (*red text*) outcomes of memory T cell–induced innate activation and cytokine production. (*a*) Microbe-specific Tmem cells facilitate rapid, tailored innate immune responses upon a reencounter with the cognate antigen. The less stringent activation of Tmem cells ensures the induction of proinflammatory innate cytokines and has been shown to promote tissue-wide protection during reinfection. Strategies to enhance memory formation may facilitate improved protection via the engagement of this axis. (*b*) Tumor antigen–specific CD4^+^ T cells activate DCs to prime CD8^+^ T cells that can then kill tumor cells. CAR-T cells might engage myeloid cells to drive immune-related adverse events and cytokine release syndrome. (*c*) Autoimmune disease is characterized by the overactivation of self-reactive T cells. Interestingly, many autoimmune conditions are also defined by high levels of innate cytokines in the absence of microbial infection. Tmem-DC cross talk through TNFSF interactions likely contributes to these innate signatures and represents a highly attractive therapeutic avenue. (*d*) Transplant rejection is primarily associated with the presence of alloreactive memory T cells. The engagement of host myeloid cells by donor T cells can result in beneficial outcomes in leukemia. However, solid organs are likely to be rejected because host memory CD4^+^ T cells, engaging DCs that present alloantigens, induce innate inflammation. The targeting of specific TNFSF members has the potential to prolong graft acceptance. Abbreviations: CAR, chimeric antigen receptor; DC, dendritic cell; PRR, pattern recognition receptor; Tmem, memory T; TNFSF, tumor necrosis factor superfamily. Figure adapted from images created with BioRender.com.

T cell–mediated activation of the innate immune system provides a protective, nonclassical means of innate immunity to circumvent pathogen evasion. On the other hand, dysregulation of these mechanisms underlies many T cell–driven pathologies, including autoimmunity. Ultimately, the innate and adaptive immune system interface represents an excellent opportunity for therapeutic targets, with applications ranging from vaccine development to cancer and autoimmune treatments (Figure 3). Critically, differential ligand-receptor expression by T cells and DCs in each context may be used to selectively target detrimental outcomes while sparing protective immunity. This remains a promising area of ongoing investigation, as described in the following sections.

### Protection from Pathogens

Despite the ability of the innate immune system to recognize widely conserved microbial features, pathogens have co-opted several strategies to circumvent this process. Through coevolution alongside host defense, microbes have developed numerous virulence factors to suppress or avoid PRR recognition and subsequent innate activation (158). Broadly, the suppressive mechanisms harnessed by virulent pathogens can be divided into two overarching themes: mitigation of PRR recognition and suppression of downstream signaling. For a more detailed overview of individual virulence factors and their mechanisms, we refer readers to other reviews (see 158 and 159). The ultimate result of these suppressive mechanisms is impaired induction of NF-κB-, MAPK-, or IRF-dependent innate immune responses, resulting in decreased APC maturation and impaired pathogen clearance.

The activation of innate responses through PRR-independent mechanisms could therefore overcome the vulnerabilities in host defenses described above. Given that memory T cells are generated under precise conditions, these cells are ideal candidates for the activation of innate immunity. Clinical evidence supports this teleological strategy. Autosomal recessive mutations in the MyD88 adaptor protein have now been reported in 22 patients worldwide (160). Not surprisingly, these patients present with severe bacterial infections early in life due to defective TLR and IL-1R family signaling. Interestingly, as these patients age, their susceptibility to such infections is significantly reduced, with no patients presenting with invasive infections past their teens (160, 161). In contrast, patients with inactivating mutations in NEMO (NF-κB essential modulator), an adaptor protein required downstream of both the TNFRSF and TLRs, experience much more severe clinical manifestations that do not improve with age (160). Given that memory T cells accumulate over time, we speculate that innate activation by T cells may serve as a critical compensatory mechanism. It is possible that memory T cells expressing cross-reactive TCRs and specificity for multiple pathogen-derived peptides (162) facilitate protective immunity through noncanonical activation of the innate immune system. The Davis group (163) previously documented the presence of CD4^+^ T cells specific to viral antigens in unexposed humans and demonstrated that immunization with influenza vaccine generates influenza-specific memory T cells with cross-reactivity to unrelated microbes. Thus, the discovery that exposure to one pathogen leads to the generation of memory T cell responses that cross-react with additional pathogens offers a likely explanation for broader vaccine effects. These findings suggest that memory T cell formation and signaling through the TNFRSF may provide a secondary system of innate activation and microbial protection independent of PRR signaling. Therefore, vaccination strategies aimed at enhancing memory T cell formation, both systemically and within local tissues, may facilitate improved clinical outcomes by elucidating the early, broad innate activation necessary for optimal pathogen control.

### CAR-T Cell Immunotherapy: Benefits and Detrimental Outcomes of Innate Immune Activation

Currently, chimeric antigen receptor (CAR)-T cells are employed primarily in the treatment of hematological malignancies (164, 165). TNFRSF proteins provide costimulatory domains for optimal signaling through the engineered CAR-T cell receptor (166). In addition, new evidence suggests that the inclusion of TNFSF ligands on engineered CAR-T cells represents a promising therapeutic avenue. CAR-T cells engineered to overexpress CD40L (CD154) showed improved effector functions and resulted in increased DC and macrophage activation, recruitment to the spleen, and infiltration of tumors (167). Similarly, CAR-T cells engineered to produce soluble agonistic CD40 antibodies led to enhancements in CAR-T cell effector functions (168). These examples clearly highlight the critical importance of membrane-bound interactions between CAR-T cells and myeloid cells to achieve optimal anticancer responses.

Despite important clinical benefits, CAR-T cell therapy is hampered by immune-related adverse events. Cytokine release syndrome (CRS) and ICANS (immune effector cell–associated neurotoxicity syndrome) are two severe negative outcomes associated with inordinate innate inflammation (169, 170). Interestingly, while CD4^+^ CAR-T cells are the primary drivers of CRS (171), IFN-γ production from CAR-T cells appears dispensable for CRS induction (171). Indeed, as opposed to therapeutic targeting of T cell–derived cytokines, tocilizumab, an IL-6 receptor–targeting monoclonal antibody, is a standard of care for CRS (172). The roles of myeloid-derived IL-6 as both a diagnostic marker and a therapeutic target imply that myeloid activation secondary to CAR-T cell expansion is a primary driver of CRS. To this end, mouse models of CRS and ICANS have correlated toxicity to the activation status of monocytic cells and found these cells to be primary producers of both IL-6 and IL-1 (173, 174). In addition to IL-6 blockade, therapies aimed at the neutralization of IL-1 signaling have proved useful in the mitigation of CRS and associated neurotoxicity in mouse models (173, 175). Interestingly, the use of CD40L-expressing CAR-T cells in these studies worsened CRS severity, further indicating that the activation of CD40^+^ myeloid cells by CAR-T cells results in excessive production of innate cytokines (173). These studies provide clinical evidence for the role of T cells in driving innate inflammatory responses. Future studies that dissect these mechanisms to prevent excessive myeloid-derived cytokine production without compromising the antitumor effects of CAR-T cell therapy will be critical to refining this approach.

### Autoimmune Disease Pathology

T cell–mediated autoimmune diseases, such as type 1 diabetes, multiple sclerosis, rheumatoid arthritis, and IBD, are major health concerns worldwide. Autoimmune diseases are distinguished from the malfunctions of the innate immune system that result in autoinflammatory diseases (176). However, autoimmune pathophysiology involves not only self-reactive T cells and B cells but also myeloid cells of the innate immune system (177). The onset of autoimmune diseases is predicated on a breach in either central or peripheral tolerance. However, the precise involvement of the innate immune system in driving both a breach in tolerance as well as disease progression remains enigmatic. While the ability of memory T cells to drive innate immune activation offers evolutionary advantages that protect against pathogens, autoimmune disease represents a detrimental consequence of this pathway. Many of the cytokines associated with autoimmune diseases are products of innate immune activation with no clear link to PRR signaling (28, 178-180).

While memory T cells are preprogrammed to secrete effector cytokines to mobilize the innate immune system, we found that physical cognate interactions between memory CD4^+^ T cells and myeloid cells drive robust innate inflammation that is completely independent of canonical PRR activation (28). We showed that members of the TNF family expressed by effector memory CD4^+^ T cells engage their cognate receptors on APCs. We observed a discrete division of labor between CD40L and TNF in dictating the quality of the inflammatory response. Innate cytokines such as IL-6 and IL-12 primarily depended on CD40L-CD40 interaction, with a minor contribution from T cell–derived TNF (24). Using an autoimmune model of regulatory T cell depletion, we found that the blocking of CD40L-CD40 interactions and the neutralization of TNF led to the mitigation of severe pathology, even in the presence of self-reactive CD4^+^ T cells (24). These data suggest that pathology in autoimmune disease was driven by self-reactive memory CD4^+^ T cell–mediated activation of the innate immune system through TNFRSF engagement (24). Additionally, the interactions of DCs with memory CD4^+^ T cells also produced large quantities of IL-1β in a manner dependent on T cell–derived TNF. IL-1β cleavage and secretion were also observed in the absence of canonical inflammasome activation and caspase-1 (28). The production of bioactive IL-1β instead resulted from caspase-8 activation and the cleavage of pro-IL-1β following Fas ligation from T cell–derived FasL (28). In a model of passive experimental autoimmune encephalomyelitis, IL-1β-, TNFR-, and Fas-deficient recipients all failed to develop diseases, suggesting that memory CD4^+^ T cell–induced IL-1β is a critical driver of autoimmune disease pathology (28). Interestingly, other researchers have shown that innate inflammation in the setting of influenza challenge was independent of TNF signaling and CD40 signaling (23), whereas we found that TNFR and CD40 played a critical role in autoreactive memory T cell–induced innate inflammation and pathology (24). While it is likely that other TNFSF family members expressed by memory CD4^+^ T cells mediate innate inflammation in the context of pathogen protection, this apparent difference in TNFSF usage in protection and pathology makes these ligand-receptor interactions especially attractive therapeutic targets.

Therapeutic immunomodulation using neutralizing antibodies against TNFRSF members on myeloid cells has been explored, specifically for the treatment of T cell–mediated autoimmune diseases, and has been discussed in depth (see 119). Inhibitors of TNF, including infliximab, adalimumab, and etanercept, are widely employed therapies for the treatment of many autoimmune disorders (181, 182). While the effectiveness of these therapies in rheumatic disease is well established, the mechanism of action in other autoimmune diseases, such as IBD, is less clear, and efficacy in patients is extremely variable (183, 184). In addition to TNF, the differential expression of TNFSF ligands on T cell subsets, as well as receptor expression on various myeloid populations, has been addressed in previous sections. We speculate that in addition to variations in autoreactive T cells, there are also disease- and tissue-specific receptor expression profiles that drive the exacerbation of innate inflammation and pathology. The highly contextual nature of TNFSF ligand-receptor expression and interactions in various disease settings warrants further investigation for the future development of precision therapeutics.

Our lab (25) recently demonstrated that memory CD4^+^ T cell interaction with myeloid cells results in double-stranded DNA breaks. The subsequent myeloid-intrinsic DNA damage response results in proinflammatory cytokine production via the activation of a noncanonical STING–TRAF6–NF-κB axis. Notably, this DNA damage response depends on cognate interactions between memory CD4^+^ T cells and myeloid cells. Because DNA damage and mutations arising from faulty repair are a hallmark of oncogenesis, we speculate that excessive interactions of autoreactive memory CD4^+^ T cells with cells presenting self-antigens might result in myeloid-specific disorders, including myelodysplastic syndrome and leukemia. Consistently, patients with autoimmunity have a predisposition to myeloproliferative neoplasms, as well as multiple myeloma and other cancers (185-188). Interestingly, other researchers recently identified a Th17 cell subpopulation that induces the transformation of intestinal epithelial cells (IECs) in the setting of chronic inflammation, resulting in adenocarcinoma (189). IEC expression of MHC-II was demonstrated decades ago, and more recent literature supports a functional role of IECs as non-conventional APCs with the ability to modulate CD4^+^ T cell responses (190, 191). The recent finding of Th17-driven oncogenesis is in line with our findings of memory CD4^+^ T cell–driven DNA damage and has wide implications for memory CD4^+^ T cell–APC cross talk as a driver of malignant transformation. In addition to which upstream molecules are involved in the initiation of DNA damage responses in interacting cells, whether these mechanisms are relevant to cells of nonmyeloid origin remains an outstanding question. Future studies are likely to determine the contribution of this mechanism to oncogenesis in the setting of autoimmunity.

### Transplant Rejection

Solid organ transplantation is a curative approach for patients with terminal organ dysfunction. The success of this approach is severely limited by host T cells specific to donor antigens, which represent approximately 1–10% of the T cell repertoire (192). Studies showing accelerated rejection of allografts in patients previously engrafted with organs from the same donor strengthen the idea that memory T cells play a vital role in transplant rejection (193). Mice previously infected with LCMV (lymphocytic choriomeningitis virus) or *Leishmania major* demonstrate accelerated allograft rejection due to the presence of cross-reactive memory T cells, also referred to as heterologous immunity (194). Although these observations suggest that preexisting memory T cells negatively affect graft survival, the precise molecular mechanisms and pathways driving rejection are not well understood. IL-12, IL-6, TNF, and IL-1β have been characterized as acute phase mediators of transplant rejection in allograft recipients (195). Perplexingly, late-phase innate proinflammatory responses have been observed more than a year after allograft transplantation. While damage-associated molecular patterns, potentially derived from transplant ischemia reperfusion injury, are proposed drivers of this innate inflammatory response, this explanation does not adequately account for the occurrence of prolonged responses that extend beyond the acute/subacute time frame (196, 197). Several lines of evidence support an alternative mechanism of innate inflammation triggered by memory CD4^+^ T cells. Alloreactive memory CD4^+^ T cell interaction with allogeneic DCs leads to DC activation and secretion of IL-1β and IL-6 (198). The production of proinflammatory cytokines critically depends on TNF-TNFR and CD40-CD40L pathways. CD4^+^ T cells also license DCs to promote the priming of alloreactive CD8^+^ T cells. The combination of memory CD4^+^ T cell–driven innate inflammation and the de novo priming of CD8^+^ T cells results in the rejection of transplanted organs (198, 199). The blockade of CD40:CD40L signaling has been shown to induce tolerance to skin allografts in mice, and this tolerance depended on the presence of IFN-γ^+^CD4^+^ T cells in the graft (200). It is likely that additional TNFSF members contribute to allograft rejection. For example, the blockade of CD28 and CD40 pathways resulted in prolonged allograft survival in Fas-deficient mice compared with Fas-proficient controls (201). Consistent with our previous observations implicating the TNFSF, specifically CD40L and FasL, in the activation of DCs by memory T cells, these pathways can be further exploited to facilitate transplant tolerance.

## CONCLUDING REMARKS

The vertebrate immune system has developed multiple strategies to recognize and respond to microbial threats. As outlined above, the innate sensing strategy is composed of at least four distinct modules that depend on the detection of conserved microbial nonself molecules or products of cellular damage. Emerging evidence suggests that memory T cells serve as microbial surrogates to induce innate inflammation independently of PRR activation. Thus, we present the cognate interaction of myeloid cells with memory T cells through the engagement of TNFSF ligands and receptors as a fifth module of innate activation (Figure 1). Why would a system that can bypass canonical self-nonself discrimination have evolved? Because naive T cells already undergo rigorous checkpoints during their initial priming, their ability to activate the innate immune system as memory T cells benefits the host in combatting the microbial evasion of innate PRR-mediated detection (Figure 2). It is quite remarkable that memory T cells of a specific lineage appear to induce innate inflammation that recapitulates responses elicited during primary infection. While T cell instruction is critical for pathogen control during reinfections, aberrant T cell instruction of the innate immune system can result in many T cell–driven pathologies, including autoimmunity, cytokine storms, and transplant rejection, as we detail (Figure 3). In each of these cases, memory T cells that are reactive to self-antigens or alloantigens or are artificially activated (as in the case of CAR-T cells) appear to engage myeloid cells, resulting in tissue damage and undesirable outcomes. Investigations over the past couple decades have led to major breakthroughs in our understanding of the innate control of adaptive immunity. The dissection of the molecular mechanisms of memory T cell–driven innate inflammation presents untapped potential for the therapeutic targeting of various T cell–mediated pathologies.
